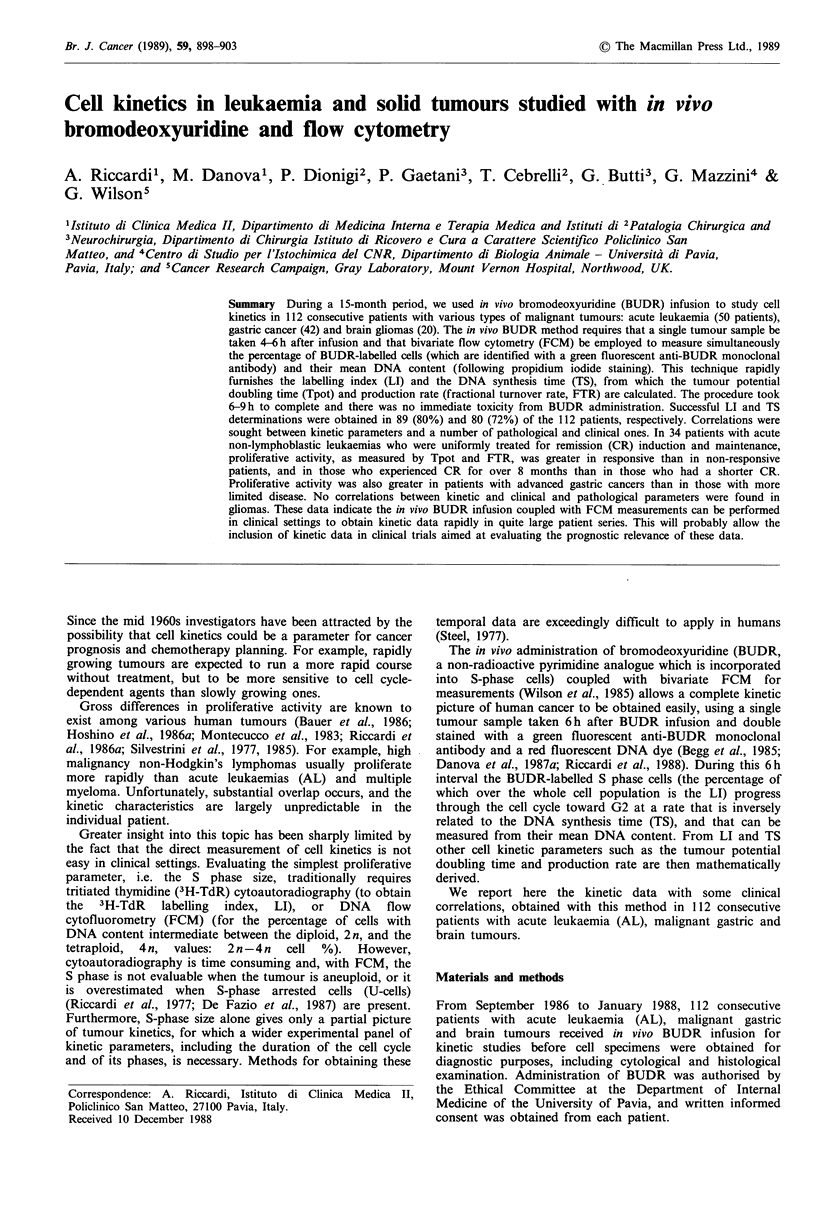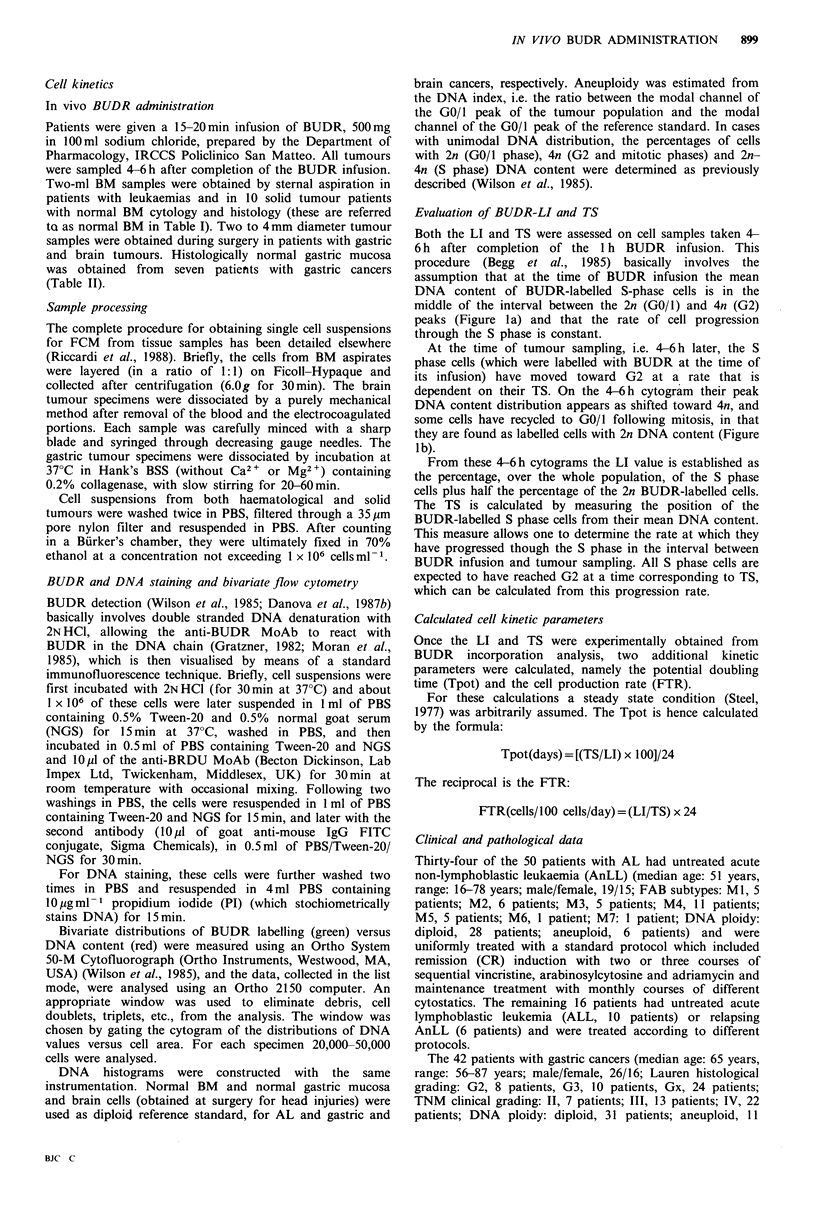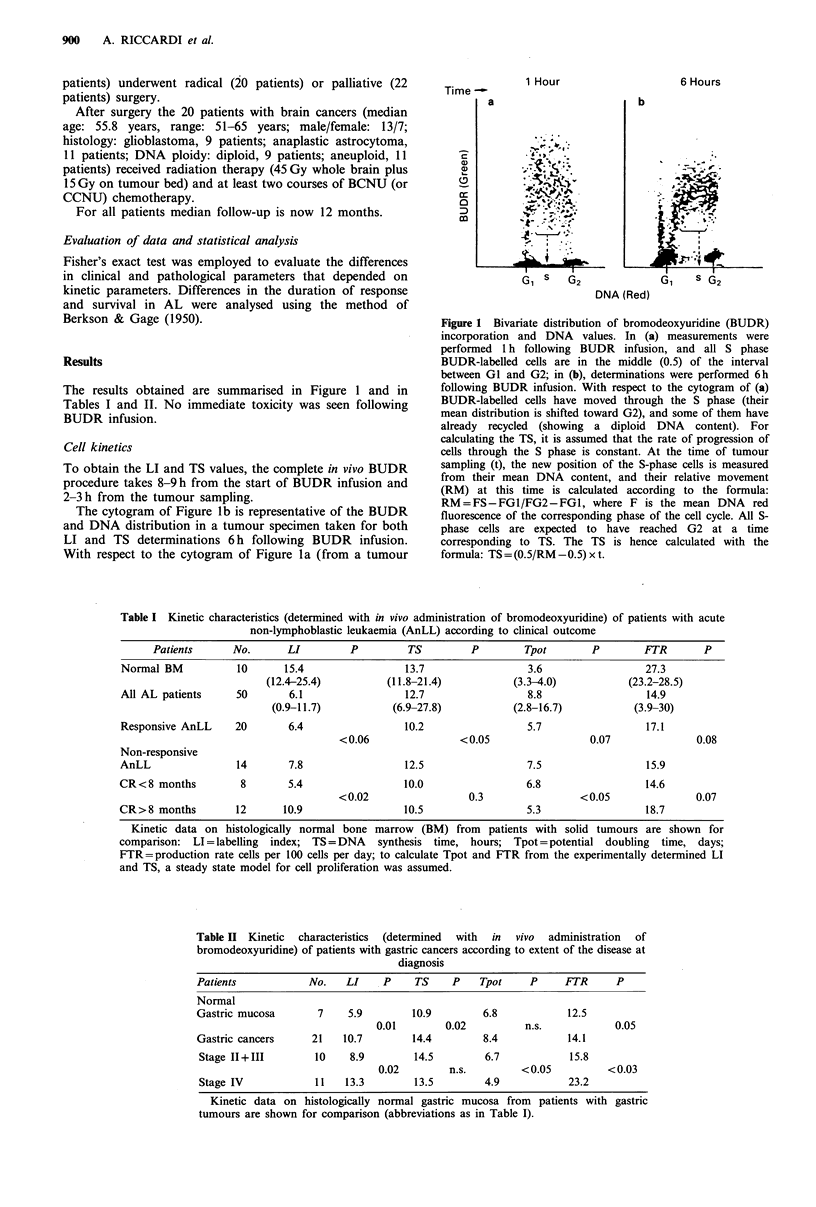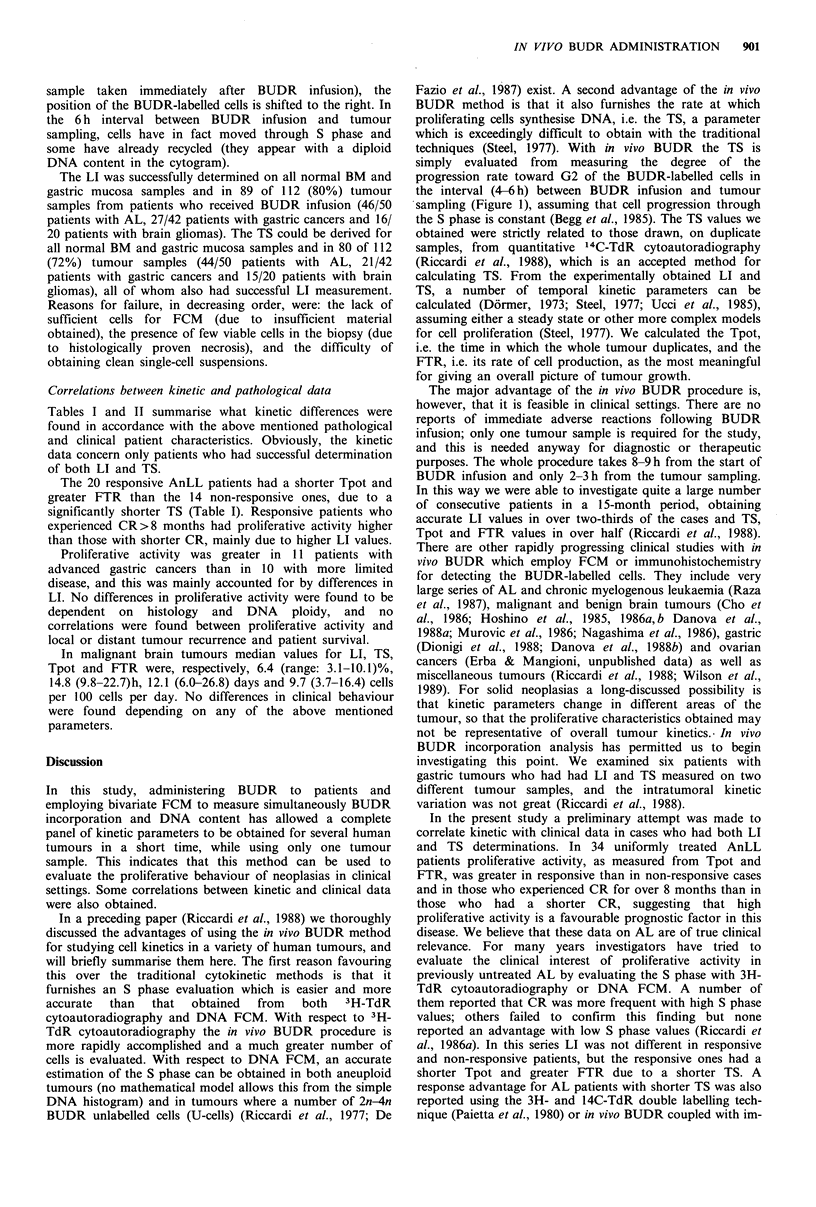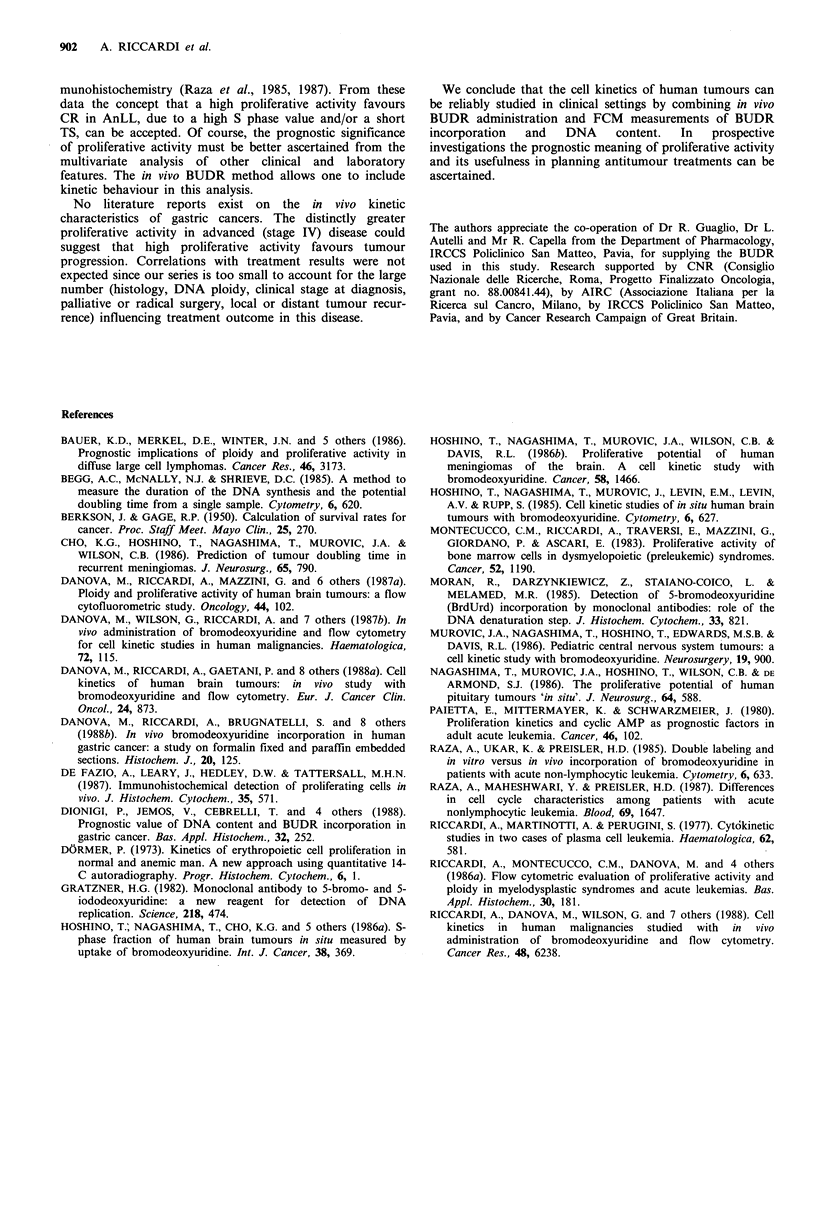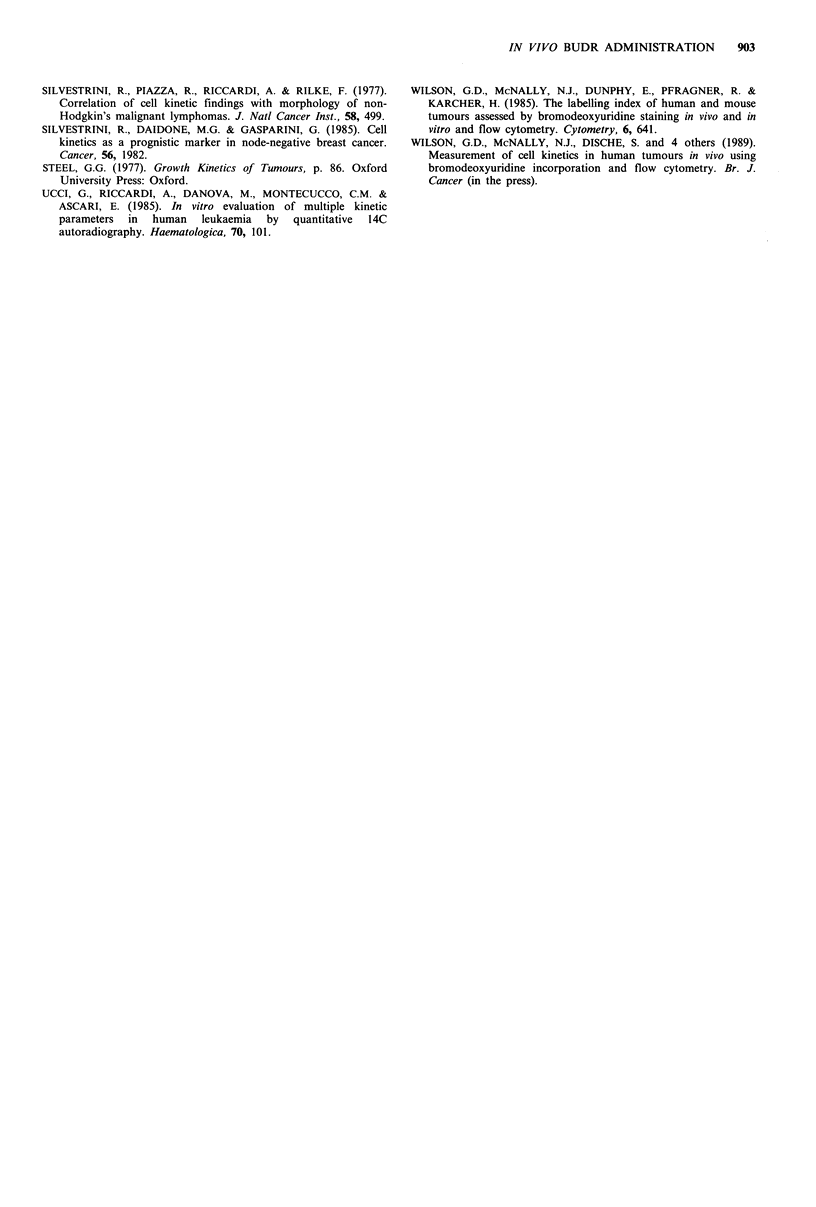# Cell kinetics in leukaemia and solid tumours studied with in vivo bromodeoxyuridine and flow cytometry.

**DOI:** 10.1038/bjc.1989.190

**Published:** 1989-06

**Authors:** A. Riccardi, M. Danova, P. Dionigi, P. Gaetani, T. Cebrelli, G. Butti, G. Mazzini, G. Wilson

**Affiliations:** Dipartimento di Medicina Interna e Terapia Medica, Policlinico San Matteo, Italy.

## Abstract

During a 15-month period, we used in vivo bromodeoxyuridine (BUDR) infusion to study cell kinetics in 112 consecutive patients with various types of malignant tumours: acute leukaemia (50 patients), gastric cancer (42) and brain gliomas (20). The in vivo BUDR method requires that a single tumour sample be taken 4-6 h after infusion and that bivariate flow cytometry (FCM) be employed to measure simultaneously the percentage of BUDR-labelled cells (which are identified with a green fluorescent anti-BUDR monoclonal antibody) and their mean DNA content (following propidium iodide staining). This technique rapidly furnishes the labelling index (LI) and the DNA synthesis time (TS), from which the tumour potential doubling time (Tpot) and production rate (fractional turnover rate, FTR) are calculated. The procedure took 6-9 h to complete and there was no immediate toxicity from BUDR administration. Successful LI and TS determinations were obtained in 89 (80%) and 80 (72%) of the 112 patients, respectively. Correlations were sought between kinetic parameters and a number of pathological and clinical ones. In 34 patients with acute non-lymphoblastic leukaemias who were uniformly treated for remission (CR) induction and maintenance, proliferative activity, as measured by Tpot and FTR, was greater in responsive than in non-responsive patients, and in those who experienced CR for over 8 months than in those who had a shorter CR. Proliferative activity was also greater in patients with advanced gastric cancers than in those with more limited disease. No correlations between kinetic and clinical and pathological parameters were found in gliomas. These data indicate the in vivo BUDR infusion coupled with FCM measurements can be performed in clinical settings to obtain kinetic data rapidly in quite large patient series. This will probably allow the inclusion of kinetic data in clinical trials aimed at evaluating the prognostic relevance of these data.


*Br. J. Cancer* (1989), **59**, 898–903 © The Macmillan Press Ltd., 1989

### Cell kinetics in leukaemia and solid tumours studied with *in vivo* bromodeoxyuridine and flow cytometry

A. Riccardi[1], M. Danova[1], P. Dionigi[2], P. Gaetani[3], T. Cebrelli[2], G. Butti[3], G. Mazzini[4] & G. Wilson[5]

[1]*Istituto di Clinica Medica II, Dipartimento di Medicina Interna e Terapia Medica and Istituti di* [2]*Patalogia Chirurgica and* [3]*Neurochirurgia, Dipartimento di Chirurgia Istituto di Ricovero e Cura a Carattere Scientifico Policlinico San Matteo, and* [4]*Centro di Studio per l'Istochimica del CNR, Dipartimento di Biologia Animale – Università di Pavia, Pavia, Italy; and* [5]*Cancer Research Campaign, Gray Laboratory, Mount Vernon Hospital, Northwood, UK.*

**Summary** During a 15-month period, we used *in vivo* bromodeoxyuridine (BUDR) infusion to study cell kinetics in 112 consecutive patients with various types of malignant tumours: acute leukaemia (50 patients), gastric cancer (42) and brain gliomas (20). The *in vivo* BUDR method requires that a single tumour sample be taken 4–6 h after infusion and that bivariate flow cytometry (FCM) be employed to measure simultaneously the percentage of BUDR-labelled cells (which are identified with a green fluorescent anti-BUDR monoclonal antibody) and their mean DNA content (following propidium iodide staining). This technique rapidly furnishes the labelling index (LI) and the DNA synthesis time (TS), from which the tumour potential doubling time (Tpot) and production rate (fractional turnover rate, FTR) are calculated. The procedure took 6–9 h to complete and there was no immediate toxicity from BUDR administration. Successful LI and TS determinations were obtained in 89 (80%) and 80 (72%) of the 112 patients, respectively. Correlations were sought between kinetic parameters and a number of pathological and clinical ones. In 34 patients with acute non-lymphoblastic leukaemias who were uniformly treated for remission (CR) induction and maintenance, proliferative activity, as measured by Tpot and FTR, was greater in responsive than in non-responsive patients, and in those who experienced CR for over 8 months than in those who had a shorter CR. Proliferative activity was also greater in patients with advanced gastric cancers than in those with more limited disease. No correlations between kinetic and clinical and pathological parameters were found in gliomas. These data indicate the *in vivo* BUDR infusion coupled with FCM measurements can be performed in clinical settings to obtain kinetic data rapidly in quite large patient series. This will probably allow the inclusion of kinetic data in clinical trials aimed at evaluating the prognostic relevance of these data.

Since the mid 1960s investigators have been attracted by the possibility that cell kinetics could be a parameter for cancer prognosis and chemotherapy planning. For example, rapidly growing tumours are expected to run a more rapid course without treatment, but to be more sensitive to cell cycle-dependent agents than slowly growing ones.

Gross differences in proliferative activity are known to exist among various human tumours (Bauer *et al.*, 1986; Hoshino *et al.*, 1986*a*; Montecucco *et al.*, 1983; Riccardi *et al.*, 1986*a*; Silvestrini *et al.*, 1977, 1985). For example, high malignancy non-Hodgkin's lymphomas usually proliferate more rapidly than acute leukaemias (AL) and multiple myeloma. Unfortunately, substantial overlap occurs, and the kinetic characteristics are largely unpredictable in the individual patient.

Greater insight into this topic has been sharply limited by the fact that the direct measurement of cell kinetics is not easy in clinical settings. Evaluating the simplest proliferative parameter, i.e. the S phase size, traditionally requires tritiated thymidine ($^3$H-TdR) cytoautoradiography (to obtain the $^3$H-TdR labelling index, LI), or DNA flow cytofluorometry (FCM) (for the percentage of cells with DNA content intermediate between the diploid, $2n$, and the tetraploid, $4n$, values: $2n-4n$ cell %). However, cytoautoradiography is time consuming and, with FCM, the S phase is not evaluable when the tumour is aneuploid, or it is overestimated when S-phase arrested cells (U-cells) (Riccardi *et al.*, 1977; De Fazio *et al.*, 1987) are present. Furthermore, S-phase size alone gives only a partial picture of tumour kinetics, for which a wider experimental panel of kinetic parameters, including the duration of the cell cycle and of its phases, is necessary. Methods for obtaining these temporal data are exceedingly difficult to apply in humans (Steel, 1977).

Correspondence: A. Riccardi, Istituto di Clinica Medica II, Policlinico San Matteo, 27100 Pavia, Italy.
Received 10 December 1988

The *in vivo* administration of bromodeoxyuridine (BUDR, a non-radioactive pyrimidine analogue which is incorporated into S-phase cells) coupled with bivariate FCM for measurements (Wilson *et al.*, 1985) allows a complete kinetic picture of human cancer to be obtained easily, using a single tumour sample taken 6 h after BUDR infusion and double stained with a green fluorescent anti-BUDR monoclonal antibody and a red fluorescent DNA dye (Begg *et al.*, 1985; Danova *et al.*, 1987*a*; Riccardi *et al.*, 1988). During this 6 h interval the BUDR-labelled S phase cells (the percentage of which over the whole cell population is the LI) progress through the cell cycle toward G2 at a rate that is inversely related to the DNA synthesis time (TS), and that can be measured from their mean DNA content. From LI and TS other cell kinetic parameters such as the tumour potential doubling time and production rate are then mathematically derived.

We report here the kinetic data with some clinical correlations, obtained with this method in 112 consecutive patients with acute leukaemia (AL), malignant gastric and brain tumours.

### Materials and methods

From September 1986 to January 1988, 112 consecutive patients with acute leukaemia (AL), malignant gastric and brain tumours received *in vivo* BUDR infusion for kinetic studies before cell specimens were obtained for diagnostic purposes, including cytological and histological examination. Administration of BUDR was authorised by the Ethical Committee at the Department of Internal Medicine of the University of Pavia, and written informed consent was obtained from each patient.

*Cell kinetics*

In vivo *BUDR administration*

Patients were given a 15–20 min infusion of BUDR, 500 mg in 100 ml sodium chloride, prepared by the Department of Pharmacology, IRCCS Policlinico San Matteo. All tumours were sampled 4–6 h after completion of the BUDR infusion. Two-ml BM samples were obtained by sternal aspiration in patients with leukaemias and in 10 solid tumour patients with normal BM cytology and histology (these are referred to as normal BM in Table I). Two to 4 mm diameter tumour samples were obtained during surgery in patients with gastric and brain tumours. Histologically normal gastric mucosa was obtained from seven patients with gastric cancers (Table II).

*Sample processing*

The complete procedure for obtaining single cell suspensions for FCM from tissue samples has been detailed elsewhere (Riccardi *et al.*, 1988). Briefly, the cells from BM aspirates were layered (in a ratio of 1:1) on Ficoll–Hypaque and collected after centrifugation (6.0*g* for 30 min). The brain tumour specimens were dissociated by a purely mechanical method after removal of the blood and the electrocoagulated portions. Each sample was carefully minced with a sharp blade and syringed through decreasing gauge needles. The gastric tumour specimens were dissociated by incubation at 37°C in Hank's BSS (without $Ca^{2+}$ or $Mg^{2+}$) containing 0.2% collagenase, with slow stirring for 20–60 min.

Cell suspensions from both haematological and solid tumours were washed twice in PBS, filtered through a 35 $\mu$m pore nylon filter and resuspended in PBS. After counting in a Bürker's chamber, they were ultimately fixed in 70% ethanol at a concentration not exceeding $1 \times 10^6$ cells $ml^{-1}$.

*BUDR and DNA staining and bivariate flow cytometry*

BUDR detection (Wilson *et al.*, 1985; Danova *et al.*, 1987*b*) basically involves double stranded DNA denaturation with 2N HCl, allowing the anti-BUDR MoAb to react with BUDR in the DNA chain (Gratzner, 1982; Moran *et al.*, 1985), which is then visualised by means of a standard immunofluorescence technique. Briefly, cell suspensions were first incubated with 2N HCl (for 30 min at 37°C) and about $1 \times 10^6$ of these cells were later suspended in 1 ml of PBS containing 0.5% Tween-20 and 0.5% normal goat serum (NGS) for 15 min at 37°C, washed in PBS, and then incubated in 0.5 ml of PBS containing Tween-20 and NGS and 10 $\mu$l of the anti-BRDU MoAb (Becton Dickinson, Lab Impex Ltd, Twickenham, Middlesex, UK) for 30 min at room temperature with occasional mixing. Following two washings in PBS, the cells were resuspended in 1 ml of PBS containing Tween-20 and NGS for 15 min, and later with the second antibody (10 $\mu$l of goat anti-mouse IgG FITC conjugate, Sigma Chemicals), in 0.5 ml of PBS/Tween-20/NGS for 30 min.

For DNA staining, these cells were further washed two times in PBS and resuspended in 4 ml PBS containing 10 $\mu g\,ml^{-1}$ propidium iodide (PI) (which stochiometrically stains DNA) for 15 min.

Bivariate distributions of BUDR labelling (green) versus DNA content (red) were measured using an Ortho System 50-M Cytofluorograph (Ortho Instruments, Westwood, MA, USA) (Wilson *et al.*, 1985), and the data, collected in the list mode, were analysed using an Ortho 2150 computer. An appropriate window was used to eliminate debris, cell doublets, triplets, etc., from the analysis. The window was chosen by gating the cytogram of the distributions of DNA values versus cell area. For each specimen 20,000–50,000 cells were analysed.

DNA histograms were constructed with the same instrumentation. Normal BM and normal gastric mucosa and brain cells (obtained at surgery for head injuries) were used as diploid reference standard, for AL and gastric and brain cancers, respectively. Aneuploidy was estimated from the DNA index, i.e. the ratio between the modal channel of the G0/1 peak of the tumour population and the modal channel of the G0/1 peak of the reference standard. In cases with unimodal DNA distribution, the percentages of cells with 2*n* (G0/1 phase), 4*n* (G2 and mitotic phases) and 2*n*–4*n* (S phase) DNA content were determined as previously described (Wilson *et al.*, 1985).

*Evaluation of BUDR-LI and TS*

Both the LI and TS were assessed on cell samples taken 4–6 h after completion of the 1 h BUDR infusion. This procedure (Begg *et al.*, 1985) basically involves the assumption that at the time of BUDR infusion the mean DNA content of BUDR-labelled S-phase cells is in the middle of the interval between the 2*n* (G0/1) and 4*n* (G2) peaks (Figure 1a) and that the rate of cell progression through the S phase is constant.

At the time of tumour sampling, i.e. 4–6 h later, the S phase cells (which were labelled with BUDR at the time of its infusion) have moved toward G2 at a rate that is dependent on their TS. On the 4–6 h cytogram their peak DNA content distribution appears as shifted toward 4*n*, and some cells have recycled to G0/1 following mitosis, in that they are found as labelled cells with 2*n* DNA content (Figure 1b).

From these 4–6 h cytograms the LI value is established as the percentage, over the whole population, of the S phase cells plus half the percentage of the 2*n* BUDR-labelled cells. The TS is calculated by measuring the position of the BUDR-labelled S phase cells from their mean DNA content. This measure allows one to determine the rate at which they have progressed though the S phase in the interval between BUDR infusion and tumour sampling. All S phase cells are expected to have reached G2 at a time corresponding to TS, which can be calculated from this progression rate.

*Calculated cell kinetic parameters*

Once the LI and TS were experimentally obtained from BUDR incorporation analysis, two additional kinetic parameters were calculated, namely the potential doubling time (Tpot) and the cell production rate (FTR).

For these calculations a steady state condition (Steel, 1977) was arbitrarily assumed. The Tpot is hence calculated by the formula:

$$\mathrm{Tpot(days)} = [(\mathrm{TS/LI}) \times 100]/24$$

The reciprocal is the FTR:

$$\mathrm{FTR(cells/100\ cells/day)} = (\mathrm{LI/TS}) \times 24$$

*Clinical and pathological data*

Thirty-four of the 50 patients with AL had untreated acute non-lymphoblastic leukaemia (AnLL) (median age: 51 years, range: 16–78 years; male/female, 19/15; FAB subtypes: M1, 5 patients; M2, 6 patients; M3, 5 patients; M4, 11 patients; M5, 5 patients; M6, 1 patient; M7: 1 patient; DNA ploidy: diploid, 28 patients; aneuploid, 6 patients) and were uniformly treated with a standard protocol which included remission (CR) induction with two or three courses of sequential vincristine, arabinosylcytosine and adriamycin and maintenance treatment with monthly courses of different cytostatics. The remaining 16 patients had untreated acute lymphoblastic leukemia (ALL, 10 patients) or relapsing AnLL (6 patients) and were treated according to different protocols.

The 42 patients with gastric cancers (median age: 65 years, range: 56–87 years; male/female, 26/16; Lauren histological grading: G2, 8 patients, G3, 10 patients, Gx, 24 patients; TNM clinical grading: II, 7 patients; III, 13 patients; IV, 22 patients; DNA ploidy: diploid, 31 patients; aneuploid, 11

patients) underwent radical (20 patients) or palliative (22 patients) surgery.

After surgery the 20 patients with brain cancers (median age: 55.8 years, range: 51–65 years; male/female: 13/7; histology: glioblastoma, 9 patients; anaplastic astrocytoma, 11 patients; DNA ploidy: diploid, 9 patients; aneuploid, 11 patients) received radiation therapy (45 Gy whole brain plus 15 Gy on tumour bed) and at least two courses of BCNU (or CCNU) chemotherapy.

For all patients median follow-up is now 12 months.

*Evaluation of data and statistical analysis*

Fisher's exact test was employed to evaluate the differences in clinical and pathological parameters that depended on kinetic parameters. Differences in the duration of response and survival in AL were analysed using the method of Berkson & Gage (1950).

### Results

The results obtained are summarised in Figure 1 and in Tables I and II. No immediate toxicity was seen following BUDR infusion.

*Cell kinetics*

To obtain the LI and TS values, the complete *in vivo* BUDR procedure takes 8–9 h from the start of BUDR infusion and 2–3 h from the tumour sampling.

The cytogram of Figure 1b is representative of the BUDR and DNA distribution in a tumour specimen taken for both LI and TS determinations 6 h following BUDR infusion. With respect to the cytogram of Figure 1a (from a tumour

**Figure 1** Bivariate distribution of bromodeoxyuridine (BUDR) incorporation and DNA values. In (**a**) measurements were performed 1 h following BUDR infusion, and all S phase BUDR-labelled cells are in the middle (0.5) of the interval between G1 and G2; in (**b**), determinations were performed 6 h following BUDR infusion. With respect to the cytogram of (**a**) BUDR-labelled cells have moved through the S phase (their mean distribution is shifted toward G2), and some of them have already recycled (showing a diploid DNA content). For calculating the TS, it is assumed that the rate of progression of cells through the S phase is constant. At the time of tumour sampling (t), the new position of the S-phase cells is measured from their mean DNA content, and their relative movement (RM) at this time is calculated according to the formula: $RM = FS - FG1/FG2 - FG1$, where F is the mean DNA red fluorescence of the corresponding phase of the cell cycle. All S-phase cells are expected to have reached G2 at a time corresponding to TS. The TS is hence calculated with the formula: $TS = (0.5/RM - 0.5) \times t$.

**Table I** Kinetic characteristics (determined with *in vivo* administration of bromodeoxyuridine) of patients with acute non-lymphoblastic leukaemia (AnLL) according to clinical outcome

| *Patients* | *No.* | *LI* | *P* | *TS* | *P* | *Tpot* | *P* | *FTR* | *P* |
|---|---|---|---|---|---|---|---|---|---|
| Normal BM | 10 | 15.4 (12.4–25.4) | | 13.7 (11.8–21.4) | | 3.6 (3.3–4.0) | | 27.3 (23.2–28.5) | |
| All AL patients | 50 | 6.1 (0.9–11.7) | | 12.7 (6.9–27.8) | | 8.8 (2.8–16.7) | | 14.9 (3.9–30) | |
| Responsive AnLL | 20 | 6.4 | <0.06 | 10.2 | <0.05 | 5.7 | 0.07 | 17.1 | 0.08 |
| Non-responsive AnLL | 14 | 7.8 | | 12.5 | | 7.5 | | 15.9 | |
| CR<8 months | 8 | 5.4 | <0.02 | 10.0 | 0.3 | 6.8 | <0.05 | 14.6 | 0.07 |
| CR>8 months | 12 | 10.9 | | 10.5 | | 5.3 | | 18.7 | |

Kinetic data on histologically normal bone marrow (BM) from patients with solid tumours are shown for comparison: LI = labelling index; TS = DNA synthesis time, hours; Tpot = potential doubling time, days; FTR = production rate cells per 100 cells per day; to calculate Tpot and FTR from the experimentally determined LI and TS, a steady state model for cell proliferation was assumed.

**Table II** Kinetic characteristics (determined with *in vivo* administration of bromodeoxyuridine) of patients with gastric cancers according to extent of the disease at diagnosis

| *Patients* | *No.* | *LI* | *P* | *TS* | *P* | *Tpot* | *P* | *FTR* | *P* |
|---|---|---|---|---|---|---|---|---|---|
| Normal Gastric mucosa | 7 | 5.9 | 0.01 | 10.9 | 0.02 | 6.8 | n.s. | 12.5 | 0.05 |
| Gastric cancers | 21 | 10.7 | | 14.4 | | 8.4 | | 14.1 | |
| Stage II+III | 10 | 8.9 | 0.02 | 14.5 | n.s. | 6.7 | <0.05 | 15.8 | <0.03 |
| Stage IV | 11 | 13.3 | | 13.5 | | 4.9 | | 23.2 | |

Kinetic data on histologically normal gastric mucosa from patients with gastric tumours are shown for comparison (abbreviations as in Table I).

sample taken immediately after BUDR infusion), the position of the BUDR-labelled cells is shifted to the right. In the 6 h interval between BUDR infusion and tumour sampling, cells have in fact moved through S phase and some have already recycled (they appear with a diploid DNA content in the cytogram).

The LI was successfully determined on all normal BM and gastric mucosa samples and in 89 of 112 (80%) tumour samples from patients who received BUDR infusion (46/50 patients with AL, 27/42 patients with gastric cancers and 16/20 patients with brain gliomas). The TS could be derived for all normal BM and gastric mucosa samples and in 80 of 112 (72%) tumour samples (44/50 patients with AL, 21/42 patients with gastric cancers and 15/20 patients with brain gliomas), all of whom also had successful LI measurement. Reasons for failure, in decreasing order, were: the lack of sufficient cells for FCM (due to insufficient material obtained), the presence of few viable cells in the biopsy (due to histologically proven necrosis), and the difficulty of obtaining clean single-cell suspensions.

*Correlations between kinetic and pathological data*

Tables I and II summarise what kinetic differences were found in accordance with the above mentioned pathological and clinical patient characteristics. Obviously, the kinetic data concern only patients who had successful determination of both LI and TS.

The 20 responsive AnLL patients had a shorter Tpot and greater FTR than the 14 non-responsive ones, due to a significantly shorter TS (Table I). Responsive patients who experienced CR > 8 months had proliferative activity higher than those with shorter CR, mainly due to higher LI values.

Proliferative activity was greater in 11 patients with advanced gastric cancers than in 10 with more limited disease, and this was mainly accounted for by differences in LI. No differences in proliferative activity were found to be dependent on histology and DNA ploidy, and no correlations were found between proliferative activity and local or distant tumour recurrence and patient survival.

In malignant brain tumours median values for LI, TS, Tpot and FTR were, respectively, 6.4 (range: 3.1–10.1)%, 14.8 (9.8–22.7)h, 12.1 (6.0–26.8) days and 9.7 (3.7–16.4) cells per 100 cells per day. No differences in clinical behaviour were found depending on any of the above mentioned parameters.

### Discussion

In this study, administering BUDR to patients and employing bivariate FCM to measure simultaneously BUDR incorporation and DNA content has allowed a complete panel of kinetic parameters to be obtained for several human tumours in a short time, while using only one tumour sample. This indicates that this method can be used to evaluate the proliferative behaviour of neoplasias in clinical settings. Some correlations between kinetic and clinical data were also obtained.

In a preceding paper (Riccardi *et al.*, 1988) we thoroughly discussed the advantages of using the *in vivo* BUDR method for studying cell kinetics in a variety of human tumours, and will briefly summarise them here. The first reason favouring this over the traditional cytokinetic methods is that it furnishes an S phase evaluation which is easier and more accurate than that obtained from both $^{3}$H-TdR cytoautoradiography and DNA FCM. With respect to $^{3}$H-TdR cytoautoradiography the *in vivo* BUDR procedure is more rapidly accomplished and a much greater number of cells is evaluated. With respect to DNA FCM, an accurate estimation of the S phase can be obtained in both aneuploid tumours (no mathematical model allows this from the simple DNA histogram) and in tumours where a number of $2n$–$4n$ BUDR unlabelled cells (U-cells) (Riccardi *et al.*, 1977; De Fazio *et al.*, 1987) exist. A second advantage of the *in vivo* BUDR method is that it also furnishes the rate at which proliferating cells synthesise DNA, i.e. the TS, a parameter which is exceedingly difficult to obtain with the traditional techniques (Steel, 1977). With *in vivo* BUDR the TS is simply evaluated from measuring the degree of the progression rate toward G2 of the BUDR-labelled cells in the interval (4–6 h) between BUDR infusion and tumour sampling (Figure 1), assuming that cell progression through the S phase is constant (Begg *et al.*, 1985). The TS values we obtained were strictly related to those drawn, on duplicate samples, from quantitative $^{14}$C-TdR cytoautoradiography (Riccardi *et al.*, 1988), which is an accepted method for calculating TS. From the experimentally obtained LI and TS, a number of temporal kinetic parameters can be calculated (Dörmer, 1973; Steel, 1977; Ucci *et al.*, 1985), assuming either a steady state or other more complex models for cell proliferation (Steel, 1977). We calculated the Tpot, i.e. the time in which the whole tumour duplicates, and the FTR, i.e. its rate of cell production, as the most meaningful for giving an overall picture of tumour growth.

The major advantage of the *in vivo* BUDR procedure is, however, that it is feasible in clinical settings. There are no reports of immediate adverse reactions following BUDR infusion; only one tumour sample is required for the study, and this is needed anyway for diagnostic or therapeutic purposes. The whole procedure takes 8–9 h from the start of BUDR infusion and only 2–3 h from the tumour sampling. In this way we were able to investigate quite a large number of consecutive patients in a 15-month period, obtaining accurate LI values in over two-thirds of the cases and TS, Tpot and FTR values in over half (Riccardi *et al.*, 1988). There are other rapidly progressing clinical studies with *in vivo* BUDR which employ FCM or immunohistochemistry for detecting the BUDR-labelled cells. They include very large series of AL and chronic myelogenous leukaemia (Raza *et al.*, 1987), malignant and benign brain tumours (Cho *et al.*, 1986; Hoshino *et al.*, 1985, 1986*a*,*b* Danova *et al.*, 1988*a*; Murovic *et al.*, 1986; Nagashima *et al.*, 1986), gastric (Dionigi *et al.*, 1988; Danova *et al.*, 1988*b*) and ovarian cancers (Erba & Mangioni, unpublished data) as well as miscellaneous tumours (Riccardi *et al.*, 1988; Wilson *et al.*, 1989). For solid neoplasias a long-discussed possibility is that kinetic parameters change in different areas of the tumour, so that the proliferative characteristics obtained may not be representative of overall tumour kinetics. *In vivo* BUDR incorporation analysis has permitted us to begin investigating this point. We examined six patients with gastric tumours who had had LI and TS measured on two different tumour samples, and the intratumoral kinetic variation was not great (Riccardi *et al.*, 1988).

In the present study a preliminary attempt was made to correlate kinetic with clinical data in cases who had both LI and TS determinations. In 34 uniformly treated AnLL patients proliferative activity, as measured from Tpot and FTR, was greater in responsive than in non-responsive cases and in those who experienced CR for over 8 months than in those who had a shorter CR, suggesting that high proliferative activity is a favourable prognostic factor in this disease. We believe that these data on AL are of true clinical relevance. For many years investigators have tried to evaluate the clinical interest of proliferative activity in previously untreated AL by evaluating the S phase with 3H-TdR cytoautoradiography or DNA FCM. A number of them reported that CR was more frequent with high S phase values; others failed to confirm this finding but none reported an advantage with low S phase values (Riccardi *et al.*, 1986*a*). In this series LI was not different in responsive and non-responsive patients, but the responsive ones had a shorter Tpot and greater FTR due to a shorter TS. A response advantage for AL patients with shorter TS was also reported using the 3H- and 14C-TdR double labelling technique (Paietta *et al.*, 1980) or *in vivo* BUDR coupled with immunohistochemistry (Raza et al., 1985, 1987). From these data the concept that a high proliferative activity favours CR in AnLL, due to a high S phase value and/or a short TS, can be accepted. Of course, the prognostic significance of proliferative activity must be better ascertained from the multivariate analysis of other clinical and laboratory features. The *in vivo* BUDR method allows one to include kinetic behaviour in this analysis.

No literature reports exist on the *in vivo* kinetic characteristics of gastric cancers. The distinctly greater proliferative activity in advanced (stage IV) disease could suggest that high proliferative activity favours tumour progression. Correlations with treatment results were not expected since our series is too small to account for the large number (histology, DNA ploidy, clinical stage at diagnosis, palliative or radical surgery, local or distant tumour recurrence) influencing treatment outcome in this disease.

We conclude that the cell kinetics of human tumours can be reliably studied in clinical settings by combining *in vivo* BUDR administration and FCM measurements of BUDR incorporation and DNA content. In prospective investigations the prognostic meaning of proliferative activity and its usefulness in planning antitumour treatments can be ascertained.

The authors appreciate the co-operation of Dr R. Guaglio, Dr L. Autelli and Mr R. Capella from the Department of Pharmacology, IRCCS Policlinico San Matteo, Pavia, for supplying the BUDR used in this study. Research supported by CNR (Consiglio Nazionale delle Ricerche, Roma, Progetto Finalizzato Oncologia, grant no. 88.00841.44), by AIRC (Associazione Italiana per la Ricerca sul Cancro, Milano, by IRCCS Policlinico San Matteo, Pavia, and by Cancer Research Campaign of Great Britain.

### References

BAUER, K.D., MERKEL, D.E., WINTER, J.N. and 5 others (1986). Prognostic implications of ploidy and proliferative activity in diffuse large cell lymphomas. *Cancer Res.*, **46**, 3173.

BEGG, A.C., McNALLY, N.J. & SHRIEVE, D.C. (1985). A method to measure the duration of the DNA synthesis and the potential doubling time from a single sample. *Cytometry*, **6**, 620.

BERKSON, J. & GAGE, R.P. (1950). Calculation of survival rates for cancer. *Proc. Staff Meet. Mayo Clin.*, **25**, 270.

CHO, K.G., HOSHINO, T., NAGASHIMA, T., MUROVIC, J.A. & WILSON, C.B. (1986). Prediction of tumour doubling time in recurrent meningiomas. *J. Neurosurg.*, **65**, 790.

DANOVA, M., RICCARDI, A., MAZZINI, G. and 6 others (1987*a*). Ploidy and proliferative activity of human brain tumours: a flow cytofluorometric study. *Oncology*, **44**, 102.

DANOVA, M., WILSON, G., RICCARDI, A. and 7 others (1987*b*). *In vivo* administration of bromodeoxyuridine and flow cytometry for cell kinetic studies in human malignancies. *Haematologica*, **72**, 115.

DANOVA, M., RICCARDI, A., GAETANI, P. and 8 others (1988*a*). Cell kinetics of human brain tumours: *in vivo* study with bromodeoxyuridine and flow cytometry. *Eur. J. Cancer Clin. Oncol.*, **24**, 873.

DANOVA, M., RICCARDI, A., BRUGNATELLI, S. and 8 others (1988*b*). *In vivo* bromodeoxyuridine incorporation in human gastric cancer: a study on formalin fixed and paraffin embedded sections. *Histochem. J.*, **20**, 125.

DE FAZIO, A., LEARY, J., HEDLEY, D.W. & TATTERSALL, M.H.N. (1987). Immunohistochemical detection of proliferating cells *in vivo*. *J. Histochem. Cytochem.*, **35**, 571.

DIONIGI, P., JEMOS, V., CEBRELLI, T. and 4 others (1988). Prognostic value of DNA content and BUDR incorporation in gastric cancer. *Bas. Appl. Histochem.*, **32**, 252.

DÖRMER, P. (1973). Kinetics of erythropoietic cell proliferation in normal and anemic man. A new approach using quantitative 14-C autoradiography. *Progr. Histochem. Cytochem.*, **6**, 1.

GRATZNER, H.G. (1982). Monoclonal antibody to 5-bromo- and 5-iododeoxyuridine: a new reagent for detection of DNA replication. *Science*, **218**, 474.

HOSHINO, T., NAGASHIMA, T., CHO, K.G. and 5 others (1986*a*). S-phase fraction of human brain tumours *in situ* measured by uptake of bromodeoxyuridine. *Int. J. Cancer*, **38**, 369.

HOSHINO, T., NAGASHIMA, T., MUROVIC, J.A., WILSON, C.B. & DAVIS, R.L. (1986*b*). Proliferative potential of human meningiomas of the brain. A cell kinetic study with bromodeoxyuridine. *Cancer*, **58**, 1466.

HOSHINO, T., NAGASHIMA, T., MUROVIC, J., LEVIN, E.M., LEVIN, A.V. & RUPP, S. (1985). Cell kinetic studies of *in situ* human brain tumours with bromodeoxyuridine. *Cytometry*, **6**, 627.

MONTECUCCO, C.M., RICCARDI, A., TRAVERSI, E., MAZZINI, G., GIORDANO, P. & ASCARI, E. (1983). Proliferative activity of bone marrow cells in dysmyelopoietic (preleukemic) syndromes. *Cancer*, **52**, 1190.

MORAN, R., DARZYNKIEWICZ, Z., STAIANO-COICO, L. & MELAMED, M.R. (1985). Detection of 5-bromodeoxyuridine (BrdUrd) incorporation by monoclonal antibodies: role of the DNA denaturation step. *J. Histochem. Cytochem.*, **33**, 821.

MUROVIC, J.A., NAGASHIMA, T., HOSHINO, T., EDWARDS, M.S.B. & DAVIS, R.L. (1986). Pediatric central nervous system tumours: a cell kinetic study with bromodeoxyuridine. *Neurosurgery*, **19**, 900.

NAGASHIMA, T., MUROVIC, J.A., HOSHINO, T., WILSON, C.B. & DE ARMOND, S.J. (1986). The proliferative potential of human pituitary tumours '*in situ*'. *J. Neurosurg.*, **64**, 588.

PAIETTA, E., MITTERMAYER, K. & SCHWARZMEIER, J. (1980). Proliferation kinetics and cyclic AMP as prognostic factors in adult acute leukemia. *Cancer*, **46**, 102.

RAZA, A., UKAR, K. & PREISLER, H.D. (1985). Double labeling and *in vitro* versus *in vivo* incorporation of bromodeoxyuridine in patients with acute non-lymphocytic leukemia. *Cytometry*, **6**, 633.

RAZA, A., MAHESHWARI, Y. & PREISLER, H.D. (1987). Differences in cell cycle characteristics among patients with acute nonlymphocytic leukemia. *Blood*, **69**, 1647.

RICCARDI, A., MARTINOTTI, A. & PERUGINI, S. (1977). Cytokinetic studies in two cases of plasma cell leukemia. *Haematologica*, **62**, 581.

RICCARDI, A., MONTECUCCO, C.M., DANOVA, M. and 4 others (1986*a*). Flow cytometric evaluation of proliferative activity and ploidy in myelodysplastic syndromes and acute leukemias. *Bas. Appl. Histochem.*, **30**, 181.

RICCARDI, A., DANOVA, M., WILSON, G. and 7 others (1988). Cell kinetics in human malignancies studied with *in vivo* administration of bromodeoxyuridine and flow cytometry. *Cancer Res.*, **48**, 6238.

SILVESTRINI, R., PIAZZA, R., RICCARDI, A. & RILKE, F. (1977). Correlation of cell kinetic findings with morphology of non-Hodgkin's malignant lymphomas. *J. Natl Cancer Inst.*, **58**, 499.

SILVESTRINI, R., DAIDONE, M.G. & GASPARINI, G. (1985). Cell kinetics as a prognistic marker in node-negative breast cancer. *Cancer*, **56**, 1982.

STEEL, G.G. (1977). *Growth Kinetics of Tumours*, p. 86. Oxford University Press: Oxford.

UCCI, G., RICCARDI, A., DANOVA, M., MONTECUCCO, C.M. & ASCARI, E. (1985). *In vitro* evaluation of multiple kinetic parameters in human leukaemia by quantitative 14C autoradiography. *Haematologica*, **70**, 101.

WILSON, G.D., McNALLY, N.J., DUNPHY, E., PFRAGNER, R. & KARCHER, H. (1985). The labelling index of human and mouse tumours assessed by bromodeoxyuridine staining *in vivo* and *in vitro* and flow cytometry. *Cytometry*, **6**, 641.

WILSON, G.D., McNALLY, N.J., DISCHE, S. and 4 others (1989). Measurement of cell kinetics in human tumours *in vivo* using bromodeoxyuridine incorporation and flow cytometry. *Br. J. Cancer* (in the press).